# Serum EBV EA-IgA and VCA-IgA antibodies can be used for risk group stratification and prognostic prediction in extranodal NK/T cell lymphoma: 24-year experience at a single institution

**DOI:** 10.1007/s00277-017-3013-y

**Published:** 2017-05-27

**Authors:** Yuhua Huang, Huilan Rao, Shumei Yan, Fang Wang, Qinian Wu, Yanfen Feng, Yujing Zhang

**Affiliations:** 1 State Key Laboratory of Oncology in South China, Collaborative Innovation Center for Cancer Medicine, Guangzhou, 510060 Guangdong People’s Republic of China; 2 Department of Pathology, Sun Yat-sen University Cancer Center, Guangzhou, 510060 Guangdong People’s Republic of China; 3 Department of Molecular Diagnosis, Sun Yat-sen University Cancer Center, Guangzhou, 510060 Guangdong People’s Republic of China; 4 Department of Radiation Oncology, Sun Yat-sen University Cancer Center, Guangzhou, 510060 Guangdong People’s Republic of China

**Keywords:** Extranodal NK/T cell lymphoma, Epstein-Barr virus, EA-IgA, VCA-IgA, Prognosis

## Abstract

Although extranodal NK/T cell lymphoma (ENKTCL) is consistently associated with Epstein-Barr virus (EBV) infection, the manifestation and prognostic value of serum EBV antibodies still remain unknown. One hundred and forty-one patients with ENKTCL were evaluated for serum EBV EA-IgA and VCA-IgA antibodies levels in the past 24 years in our institution. Their correlation with clinicopathological features, plasma EBV DNA load, and patients’ outcomes was analyzed. EBV EA-IgA ≥1:10 and VCA-IgA ≥1:160 were found in 18.4 and 16.3% of patients, respectively. They correlated with adverse ENKTCL profile and inferior overall survival (OS) and progression-free survival (PFS). EA-IgA ≥1:10 was an independent prognostic factor on OS (RR = 2.276, *p* = 0.008) and associated with lower complete response (CR) rate (34.8 vs 70.6%, *p* = 0.001) and higher relapse rate in CR patients (62.5 vs 34.7%, *p* = 0.016). In subgroup analysis, both EA-IgA ≥1:10 and VCA-IgA ≥1:160 significantly correlated with inferior OS and PFS in patients with stage I/II, IPI score 0–1, plasma EBV DNA (+), and CR. Patients with plasma EBV DNA (+) and EA-IgA ≥1:10 (or VCA-IgA ≥1:160) had significantly shorter periods of OS and PFS in comparison with other corresponding groups. Elevated serum EBV EA-IgA and VCA-IgA levels were related to adverse ENKTCL profile and correlated with poor treatment response, early relapse, and poor prognosis in patients with ENKTCL. These findings provide convincing evidence for the use of serum EBV EA-IgA and VCA-IgA antibodies for risk group stratification and prognostic prediction in ENKTCL.

**Electronic supplementary material:**

The online version of this article (doi:10.1007/s00277-017-3013-y) contains supplementary material, which is available to authorized users.

## Introduction

Extranodal NK/T cell lymphoma (ENKTCL) is a distinct subtype of non-Hodgkin lymphoma, which is much more prevalent in Asian and Hispanic population compared with Western population [1, 2]. It is consistently associated with Epstein-Barr virus (EBV) infection, irrespective of the ethnic origin [3, 4]. EBV shows a type II latency pattern of gene expression in ENKTCL and could be detected by EBER in situ hybridization in the tumor cells [5]. EBV serology in patients with nasopharyngeal carcinoma (NPC) has been widely investigated [6–11]. The two most widely tested EBV-related antibodies, IgA against early antigen antibody (EA-IgA) and IgA against viral capsid antigen IgA (VCA-IgA), have been widely used for assisting in diagnosis and predicting the prognosis of NPC [8, 12, 13]. On the other hand, little is known about EBV serology in ENKTCL patients up to date. Although we previously observed that a part of patients with ENKTCL had positive serum EBV EA-IgA and VCA-IgA antibodies in a small cohort (*n* = 12) [14], the clinical significance had not been explored due to the limited cases. To clarify the overall clinical significance of elevated serum EBV EA-IgA and VCA-IgA antibodies in ENKTCL, an in-depth study with a large cohort is essential to provide more definitive evidence.

ENKTCL usually shows aggressive behavior with a poor prognosis [1]. Although chemoradiotherapy and non-anthracycline-based chemotherapy have been shown to improve outcome [15, 16], treatment failures occur in patients with any stage of disease [17]. Routine pathologic and immunophenotypic evaluations are insufficient to predict the clinical outcome in ENKTCL patients, although several clinicopathological features, including International Prognostic Index (IPI), Korea Prognostic Index (KPI), Ki67 Proliferative Index, and serum beta2-microglobin, predict the prognosis [18–20]. The lack of novel prognostic markers has created significant challenges in treatment selection for the heterogeneous clinical behavior of ENKTCL, particularly for patients with stage IE/IIE disease [15, 21–23]. Therefore, novel prognostic predictors are needed to help stratify the patients in the high-risk group and it is better to improve the treatment planning of the patients with worse survivals. In recent years, plasma EBV DNA has been investigated and found to be associated with poor outcome in patients with ENKTCL [20, 24, 25]. To the best of our knowledge, the prognostic value of serum EBV EA-IgA and VCA-IgA antibodies has never been explored in ENKTCL. Therefore, we retrospectively analyze serum EBV EA-IgA and VCA-IgA antibodies in a large and well-characterized series of ENKTCL. The relationship between serum EBV EA-IgA and VCA-IgA antibodies levels and the clinicopathological features, plasma EBV DNA load, treatment response, and outcome data were analyzed thoroughly.

## Methods

### Patients and clinicopathological data

We retrospectively reviewed the medical records of 141 patients newly diagnosed as ENKTCL with serum EBV EA-IgA and VCA-IgA data at Sun Yat-sen University Cancer Center between December 1990 and December 2014. The inclusion criteria of this retrospective study were as follows: (a) the morphology and immunophenotype were in accordance with the World Health Organization (WHO, 2008) classification [26], showing cytoplasmic CD3ε+, CD56+/−, cytotoxic markers+, and EBER+ (Fig. S1); (b) previously untreated; (c) available data of serum EBV EA-IgA and VCA-IgA level at diagnosis; (d) adequate clinical and followed-up data; (e) no previous malignant tumor or second primary tumor; and (f) aggressive NK-cell leukemia and chronic active EBV (CAEVB) disease-type T/NK systemic lymphoproliferative diseases (LPD) were excluded. The study was approved by the Institutional Review Board of Sun Yat-sen University Cancer Center, and all patients provided written informed consent for the collection and publication of their medical information at the first visit to our center.

The following clinical data were collected for analysis: age, gender, systemic B symptoms, primary site, involved sites, Eastern Cooperative Oncology Group performance status (ECOG PS), serum lactate dehydrogenase (LDH) level, regional lymphadenopathy, bone marrow examinations, Ann Arbor stage, date of diagnosis, treatment modality, treatment response, date of last follow-up, progression, and survival status. In addition, the IPI (age, performance status, stage, LDH value, extranodal involvement sites) was also evaluated. The primary site of the tumor was classified into upper aerodigestive tract (UAT) and non-UAT [27].

### Serological tests for EBV VCA-IgA and EA-IgA antibodies

Serum samples were collected from patients with ENKTCL at the time of initial diagnosis. Immunoenzymatic assay (IEA) was applied to detect serum antibodies against EBV VCA-IgA and EA-IgA. IEAs were prepared from the B95 cell line for VCA and the Raji cell line for EA. Plasma samples were screened at a dilution of 1:10, followed by twofold serial dilutions. The antibody titer was the reciprocal of the highest dilution clearly showing a brown color within 15% of the cells. Levels of VCA-IgA and EA-IgA were determined by titration, with the cut-off values set at 1:40 for VCA-IgA and 1:10 for EA-IgA [28].

### Plasma EBV-DNA quantification

Plasma samples were collected from 57 out of 141 patients simultaneously before initiation of therapy. Total plasma cell-free DNA was isolated using the QIAamp Blood Mini Kit (QIAgen, Inc., Valencia, CA, USA) according to the “blood and body fluid protocol” as recommended by the manufacturer. The real-time quantitative PCR system was developed for plasma EBV DNA detection toward the BamHI-W region. The designs of amplification primers were as previously reported [25]. EBV-negative healthy volunteers were used as negative controls, and a no template control was run on each plate as a blank control. The results were expressed as the number of copies of EBV per milliliter (mL) of plasma. We chose 0 copy/mL as the cut-off value for EBV-DNA level.

### Treatment response evaluation

Treatment response was assessed according to the International Working Group Recommendations for Response Criteria for NHL [19]. Overall survival (OS) was measured from the date of diagnosis until the date of death from any cause or the last follow-up. Progression-free survival (PFS) was calculated from the date of diagnosis to the date of disease progression, relapse, and death from any cause or the last follow-up.

### Statistical analysis

The correlation between pretreatment serum EBV EA-IgA and VCA-IgA level and clinical features was analyzed by the chi-square or Fisher’s exact test. Survival analysis was performed using the Kaplan-Meier method, and comparisons were calculated using the log-rank test. Multivariate analysis was used to estimate the prognostic impact of different variables in OS and PFS using the Cox regression model. The potential risk of age, Ann Arbor stage, B symptoms, LDH level, IPI score, ECOG PS score, regional lymph node involvement, extranodal involvement sites, serum EA-IgA, and VCA-IgA level was analyzed by forward stepwise Cox proportional hazard regression model. Differences between the results from comparative tests were considered significant if the two-sided *p* value was <0.05. All analyses were performed with SPSS software (version 19.0 for window; Statistical Product and Service Solutions; IBM).

## Results

### Patient’s clinicopathological characteristics

Based on the inclusion criteria, a total of 141 ENKTCL were included into this cohort. The clinicopathological characteristics of the patients are shown in Table 1. Median age at diagnosis was 43 years old (range, 18–80) with 23 patients (16.3%) being older than 60 years old. There was a male predominance in this series, with a male-to-female ratio of 2.3:1. Seventy patients (70/139, 50.4%) presented with B symptoms. The majority (89.4%) of patients had stage I/II disease, and most patients (67.4%) were categorized to low-risk group (IPI = 0–1) according to IPI system. Only 7.4% of patients had poor performance status (ECOG score >1). Elevated LDH level was observed in 24.6% of cases. The UAT as the primary site was found in 134 cases (95.0%); among these cases, 88 cases were in nasal cavity and 46 cases were in Waldeyer ring. Non-UAT as the primary site was found in seven cases (5%), including the gingiva (two cases), gastrointestinal tract (two cases), mandible (two cases), and skin (one case). Sixty-four patients (45.4%) have regional lymph node involvement. Thirty-five (61.4%) out of 57 available cases had positive pretreatment EBV-DNA.

**Table 1:** Pretreatment serum EBV VCA-IgA and EA-IgA status of patient with ENKTCL stratified by clinicopathological features

Characteristics	EA-IgA			VCA-IgA		
	<1:10 *n* (%)	≥1:10 *n* (%)	*p* value	<1:160 *n* (%)	≥1:160 *n* (%)	*p* value
Patients	115(81.6)	26(18.4)		118(83.7)	23(16.3)	
Age						
≤60	98(83.1)	20(16.9)	0.301	103(87.3)	15(12.7)	0.026
>60	17(73.9)	6(26.1)		15(65.2)	8(34.8)	
Gender						
Male	81(82.7)	17(17.3)	0.613	86(87.8)	12(12.2)	0.048
Female	34(79.1)	9(20.9)		32(74.4)	11(25.6)	
Ann Arbor stage						
I/II	107(84.9)	19(15.1)	0.008	109(86.5)	17(13.5)	0.018
III/IV	8(53.3)	7(46.7)		9(60.0)	6(40.0)	
B symptoms						
Absence	61(87.1)	9(12.9)	0.075	63(90.0)	7(10.0)	0.058
Presence	52(75.4)	17(24.6)		54(78.3)	15(21.7)	
LDH						
Normal	87(83.7)	17(16.3)	0.190	91(87.5)	13(12.5)	0.022
Elevated	25(73.5)	9(26.5)		24(70.6)	10(29.4)	
IPI score						
0–1	80(86.0)	13(14.0)	0.036	84(90.3)	9(9.7)	0.002
2–5	32(71.1)	13(28.9)		31(68.9)	14(31.1)	
ECOG PS score						
0–1	103(82.4)	22(17.6)	0.393	106(84.8)	19(15.2)	0.209
2–5	7(70.0)	3(30.0)		7(70.0)	3(30.0)	
Primary tumor site						
UAT	109(81.3)	25(18.7)	1.000	112(83.6)	22(16.4)	1.000
Non-UAT	6(85.7)	1(14.3)		6(85.7)	1(14.3)	
Regional LN involvement						
No	71(92.2)	6(7.8)	0.000	70(90.9)	7(9.1)	0.011
Yes	44(68.8)	20(31.3)		48(75.0)	16(25.0)	
Extranodal sites						
< 2	105(84.0)	20(16.0)	0.035	108(86.4)	17(13.6)	0.019
≥ 2	9(60.0)	6(40.0)		9(60.0)	6(40.0)	
Pretreatment EBV-DNA						
Negative	22(100.0)	0(0.0)	0.002	22(100.0)	0(0.0)	0.004
Positive	23(65.7)	12(34.3)		24(68.6)	11(31.4)	
Treatment response						
CR	72(90.0)	8(10.0)	0.001	70(87.5)	10(12.5)	0.086
Non-CR	30(66.7)	15(33.3)		34(75.6)	11(24.4)	

B symptoms include unexplained fever with temperature above 38 °C, night sweating or weight loss more than 10% within 6 months. *LDH* lactate dehydrogenase, *IPI* International Prognostic Index, *ECOG PS* Eastern Cooperative Oncology Group performance status, *UAT* upper aerodigestive tract, *CR* complete response

### Association of serum EBV EA-IgA and VCA-IgA antibodies levels with clinicopathological features

Overall, positive EA-IgA was detected in 18.4% of patients and 41.1% for VCA-IgA, with geometric mean titers (GMT) of 1:14.4 and 1:58.3, respectively. Highest serum EA-IgA and VCA-IgA titers observed in this cohort were 1:160 and 1:1280, respectively. VCA-IgA was further classified into two different levels using 1:160 as cut-off point. Hence, 16.3% of patients had high level VCA-IgA (≥1:160), while 83.7% for low-level VCA-IgA (<1:160).

As listed in Table 1, both serums EA-IgA ≥1:10 and VCA-IgA ≥1:160 were significantly associated with advanced stage disease (*p* = 0.008 and *p* = 0.018, respectively), IPI score ≥ 2 (*p* = 0.036 and *p* = 0.002, respectively), regional lymph node involvement (*p* < 0.001 and *p* = 0.011, respectively), and extranodal involvement sites >1 (*p* = 0.035 and *p* = 0.019, respectively). In addition, patients with serum VCA-IgA ≥1:160 at diagnosis were more likely in female (*p* = 0.048), age > 60 years (*p* = 0.026) and elevated LDH group (*p* = 0.022). No significant association was found for the other clinicopathological parameters, including B symptoms, ECOG PS score, and primary tumor site.

### Association of serum EBV EA-IgA and VCA-IgA level with plasma EBV DNA load

Positivity for plasma EBV DNA was present in 35 out of 57 (61.4%) available cases. The relationship between serum EBV EA-IgA and VCA-IgA and plasma EBV DNA were examined. Both EA-IgA ≥1:10 and VCA-IgA ≥1:160 correlated positively with plasma EBV DNA (*p* = 0.002 and *p* = 0.004, respectively). Among EBV DNA-positive patients, 34.3 (12/35) and 31.4% (11/35) of cases were found to have EA-IgA ≥1:10 and VCA-IgA ≥1:160, respectively; while no case with EA-IgA ≥1:10 or VCA-IgA ≥1:160 was found among EBV DNA-negative patients.

### Effect of serum EBV EA-IgA and VCA-IgA antibody level on treatment response and subsequent relapse

Forty-four patients (31.2%) received chemotherapy alone and nine (6.4%) underwent radiotherapy alone, while 86 patients (61.0%) had chemotherapy followed by radiotherapy, two patients (1.4%) received best supported care. The first-line regimens of chemotherapy were as follows: 53 patients received CHOP (cyclophosphamide, doxorubicin, vincristine, and prednisone) or CHOP-like, 23 patients received EPOCH (etoposide, doxorubicin, vincristine, cyclophosphamide, prednisone), 19 patients received GELOX (gemcitabine, L-asparaginase, oxaliplatin), 14 patients were treated with an alternating triple therapy ((CHOP-B, IMVP-16, and DHAP): CHOP-B (cyclophosphamide, doxorubicin, vincristine, prednisone, and bleomycin), IMVP-16 (ifosfamide, etoposide, methotrexate), DHAP (dexamethasone, cytarabine, cisplatin)), and others received GEMOX (gemcitabine, oxaliplatin) and SMILE (dexamethasone, methotrexate, ifosfamide, L-asparaginase, etoposide). At the end of treatment, 80 of 125 (64%) patients with response evaluation got complete response (CR).

**Fig. 1:**
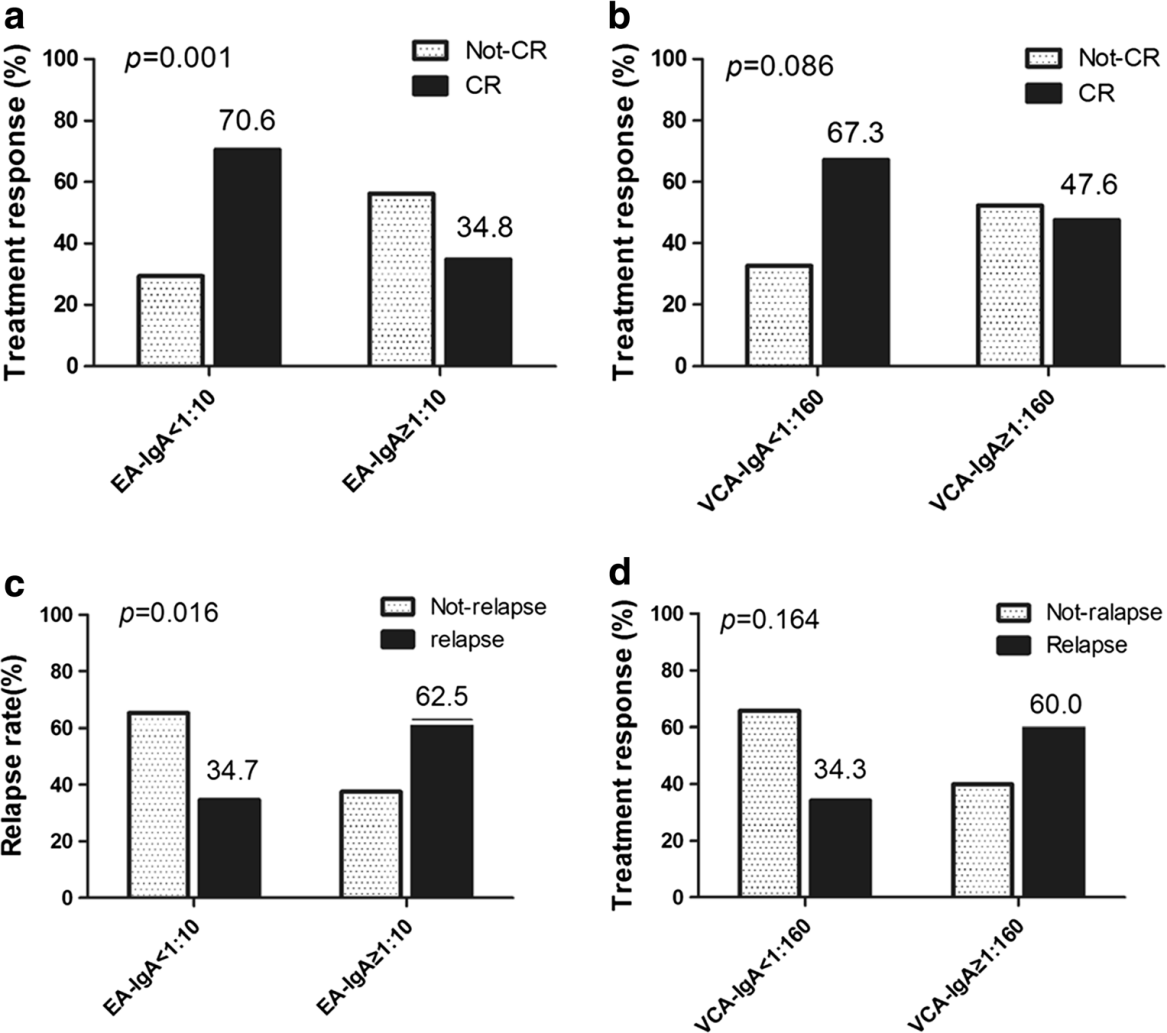
Effect of serum EBV EA-IgA and VCA-IgA antibody level on treatment response. **a** Patients with serum EA-IgA ≥1:10 had significantly lower CR rate. **b** Serum VCA-IgA ≥1:160 was related to lower CR rate with borderline significance. **c** EA-IgA ≥1: 10 was significantly associated with higher relapse rate in CR patients. **d** VCA-IgA ≥1: 160 was related to higher relapse rate in CR patients, but the difference did not reach statistical significance

As is shown in Table 1, patients with serum EA-IgA ≥1:10 had significantly lower CR rate (34.8%, 8/23 vs 70.6%, 72/102, *p* = 0.001) (Fig. 1). Serum VCA-IgA ≥1:160 was also related to lower CR rate with borderline significance (47.6%, 10/21 vs 67.3%, 70/104, *p* = 0.086). To study the prognostic impacts of serum EBV EA-IgA and VCA-IgA antibody level on subsequent relapses in CR patients, we compared the relapse rates between patients with different levels of serum EA-IgA and VCA-IgA antibodies. Interestingly, pretreatment serum EA-IgA ≥1:10 showed significant impact on subsequent tumor relapse. Patients with serum EA-IgA ≥1:10 had significantly higher relapse rate (62.5%, 5/8 vs 34.7%, 25/72, *p* = 0.016) (Fig. 1). Patients with pretreatment serum VCA-IgA ≥1:160 also seemed to have higher relapse rate (60.0%, 6/10 vs 34.3%, 24/70), but the difference did not reach statistical significance (*p* = 0.164).

### Survival and prognostic factors

At a median follow-up time of 28 months (range 1–157), 75 patients had disease progression or relapse at a median of 6.7 months (1–125.6), of whom 53 patients died of tumor progression at a median of 8.3 months (1–83.8). The 3-year PFS rate and OS rate were 48 and 60%, respectively.

**Fig. 2:**
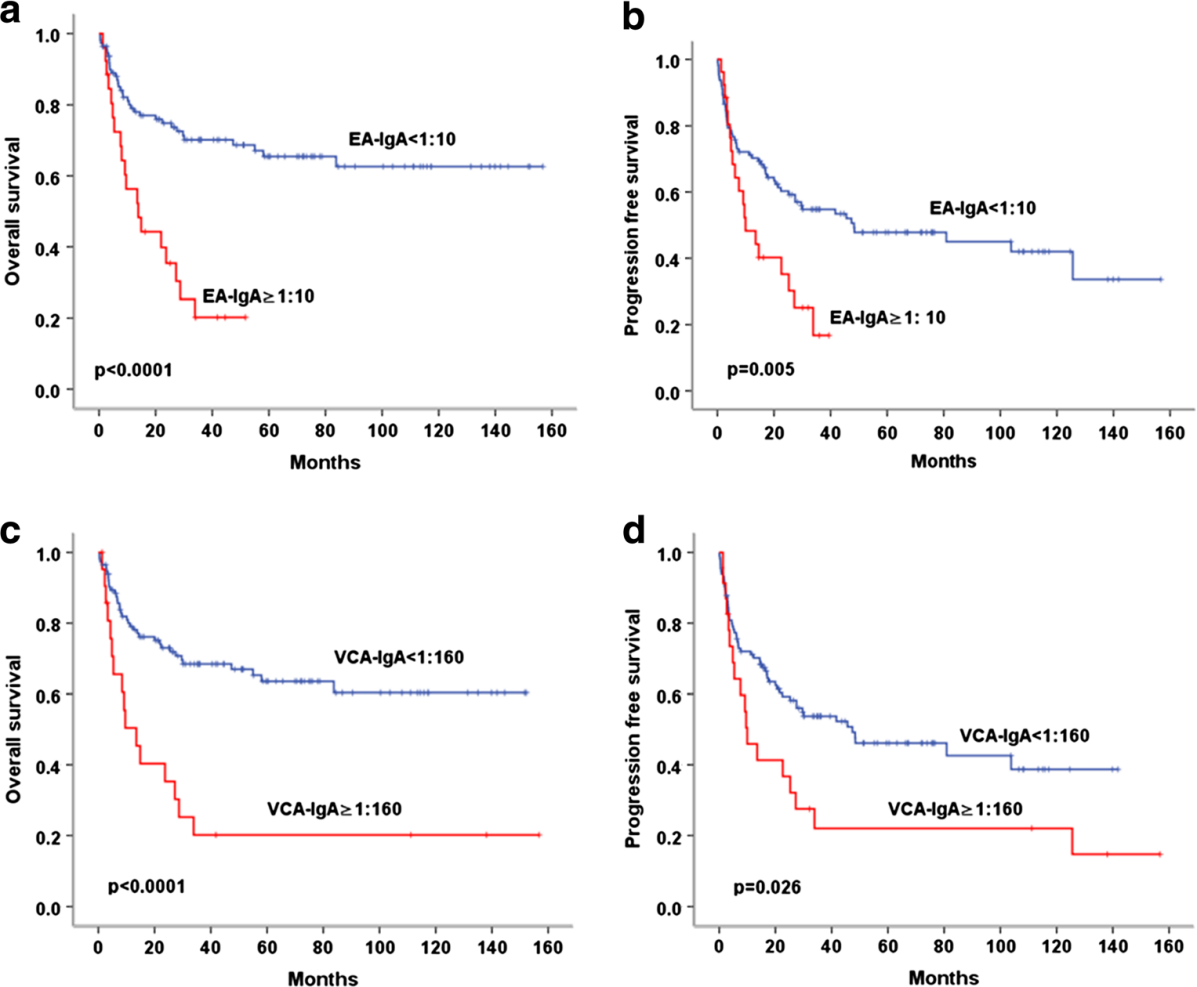
Survival analysis of OS and PFS in the whole cohort of 141 patients with ENKTCL according to serum EA-IgA and VCA-IgA level. **a**, **b** Patients with serum EA-IgA ≥1:10 had inferior OS and PFS; **c**, **d** Serum VCA-IgA ≥1:160 significantly related to inferior OS and PFS

In univariate survival analysis, age (>60), Ann Arbor stage (III/IV), LDH (elevated), IPI score (≥2), ECOG PS score (≥2), regional lymph node involvement, extronodal involvement sites (≥2), pretreatment EBV-DNA level (positive), and treatment response (non-CR) significantly correlated with both inferior OS and PFS (both *p* < 0.05). Patients with pretreatment serum EA-IgA ≥1:10 had inferior OS (3-year OS, 20 vs 70%; *p* < 0.0001) and PFS (3-year PFS, 14 vs 50%; *p* = 0.005). VCA-IgA ≥1:160 also associated with worse OS (3-year OS, 21 vs 68%; *p* < 0.0001) and PFS (3-year PFS, 22 vs 53%; *p* = 0.026) (Fig. 2).

The possible prognostic variables (age, Ann Arbor stage, B symptoms, LDH level, IPI score, ECOG PS score, regional lymph node involvement, extranodal involvement sites, serum EA-IgA, and VCA-IgA) were included in the multivariate analysis. In the forward conditional Cox regression model, serum EA-IgA ≥1:10 was found to be a strong unfavorable predictor of OS in patients with ENKTCL (RR = 2.276, 95% CI = 1.240–4.178, *p* = 0.008), independent of IPI score, and regional lymph node involvement (Table 2).

**Table 2:** Multivariate analysis for OS and PFS in patients with ENKTCL

Parameters	Relative risk (RR)	95% confidence index (CI)		*p* value
		Lower	Upper	
Overall survival (OS)				
IPI score: 2–5	4.466	2.502	7.974	0.000
Regional LN involvement: yes	1.852	1.028	3.337	0.040
EA-IgA: ≥1:10	2.276	1.240	4.178	0.008
Progression-free survival (PFS)				
IPI score: 2–5	2.744	1.692	4.450	0.000
Regional LN involvement: yes	1.978	1.978	1.220	0.006

*OS* overall survival, *PFS* progression-free survival, *ENKTCL* extranodal NK/T cell lymphoma, *IPI* International Prognostic Index

### Subgroup analysis

**Fig. 3:**
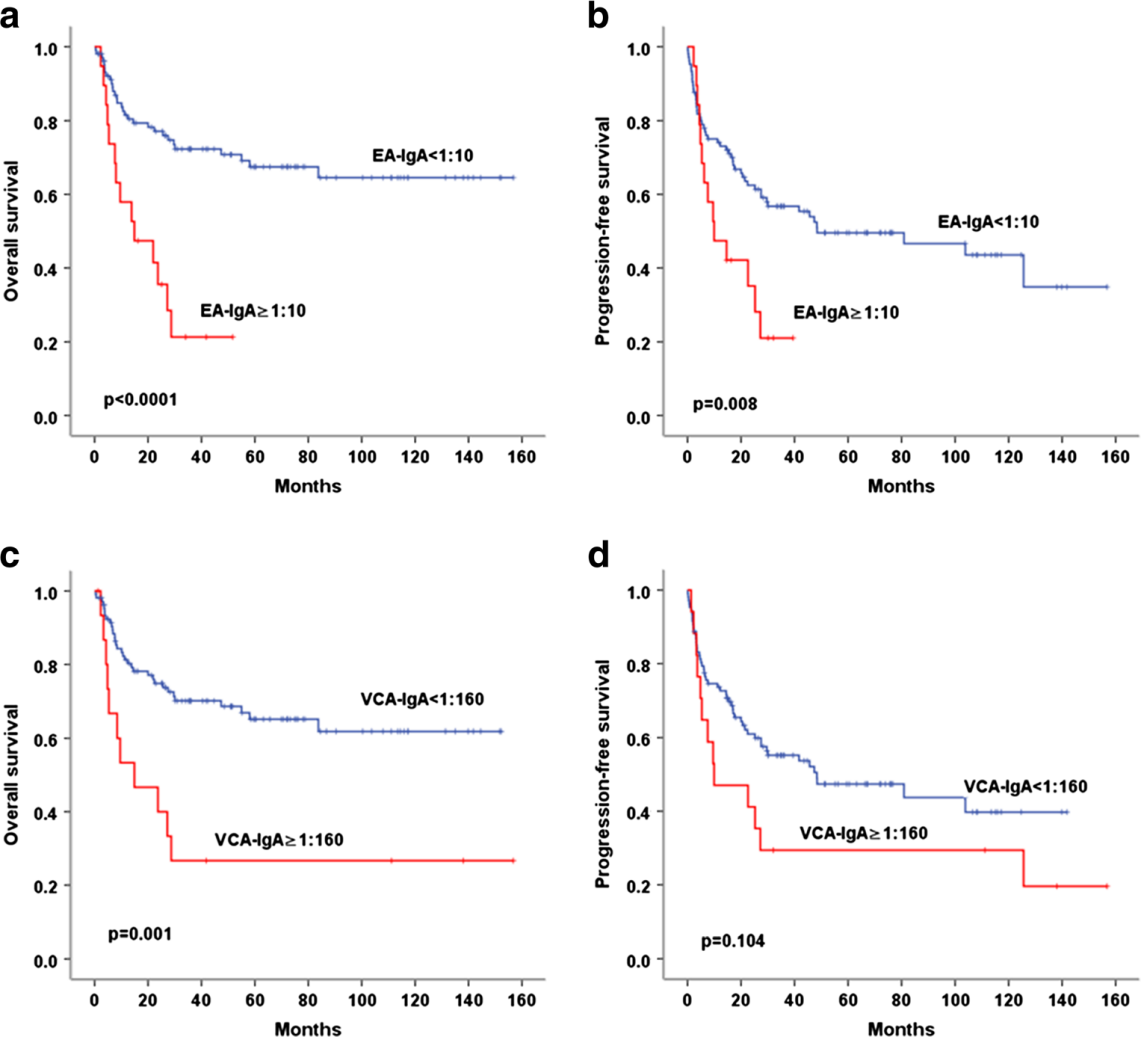
Comparison of OS and PFS in patients with stage I / II according to serum EA-IgA and VCA-IgA level. **a**, **b** Serum EA-IgA ≥1:10 was significantly associated with poor PFS and OS in patients with stage I /II. **c** Serum VCA-IgA ≥1:160 was significantly related to poorer OS in patients with stage I/II. **d** Serum VCA-IgA ≥1:160 related to poorer PFS in patients with stage I/II, but the difference did not reach statistical significance

In subgroup analysis, pretreatment serum EA-IgA and VCA-IgA level could distinguish those with poor outcomes from those with favorable outcomes in patients with early stage (stage I/II). Serum EA-IgA ≥1:10 was associated with poor PFS and OS in patients with stage I/II (both *p* < 0.0001). Serum VCA-IgA ≥1:160 was also significantly related to poorer OS (*p* = 0.001), but not PFS (*p* = 0.104) (Fig. 3). In contrast, in patients with stage III/IV, neither EA-IgA nor VCA-IgA was significantly associated with outcome.

Grouping by the IPI score, both EA-IgA ≥1:10 and VCA-IgA ≥1:160 were found to significantly affect OS (*p* = 0.001 and *p* = 0.004, respectively) in patients with IPI score 0–1 (Fig. S2), but not PFS (either *p* > 0.005). Neither EA-IgA nor VCA-IgA level affected OS or PFS in patients with IPI score 2–5.

**Fig. 4:**
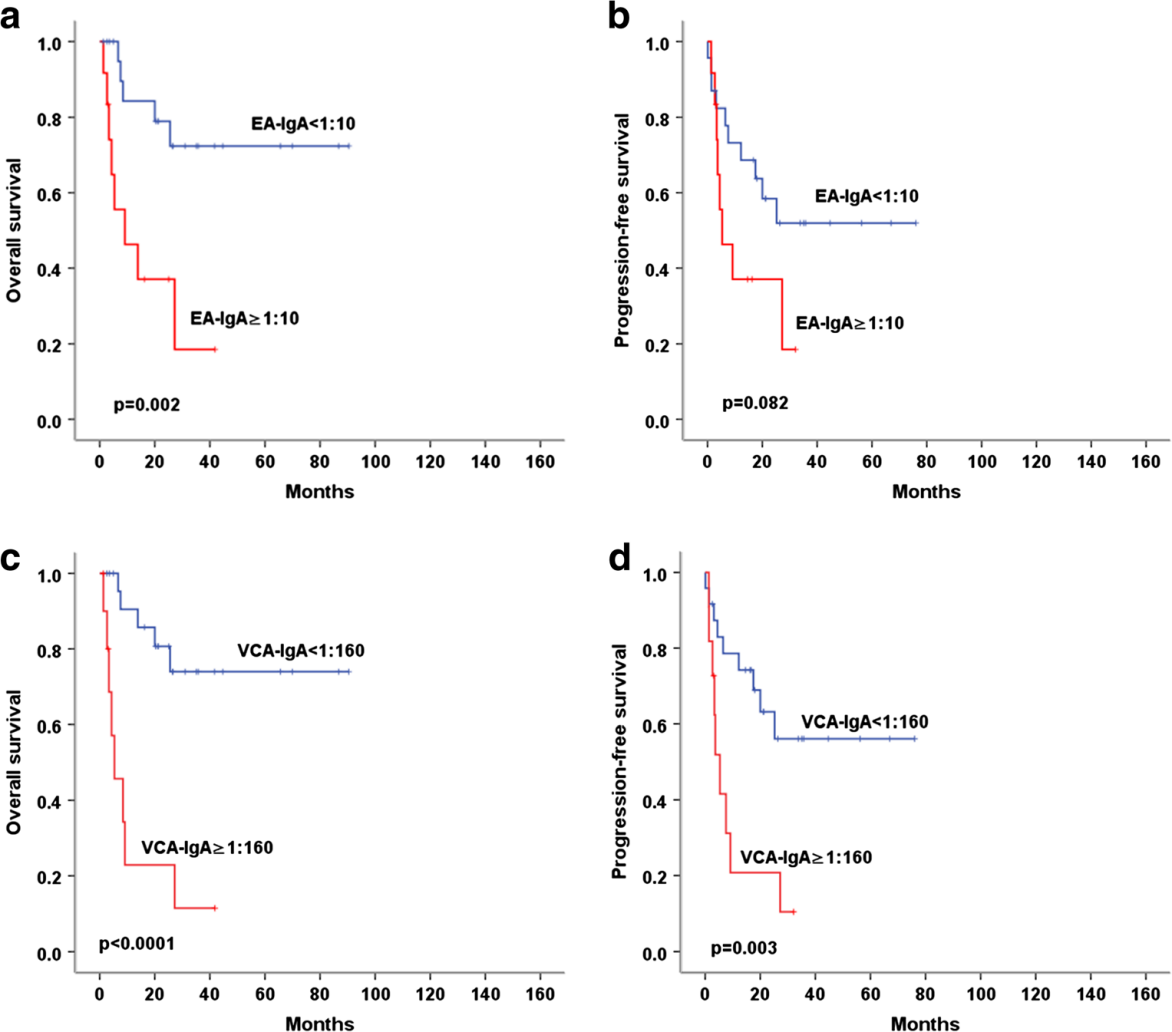
Comparison of OS and PFS in patients with positive EBV-DNA according to serum EA-IgA and VCA-IgA level. **a**, **b** EA-IgA ≥1:10 was related to inferior OS with statistical significance and was related to poorer PFS with borderline significance in patients with positive pretreatment EBV-DNA. **c**, **d** Serum VCA-IgA ≥1:160 was significantly related to inferior OS and PFS in patients with positive pretreatment EBV-DNA

In patients with positive pretreatment EBV-DNA, EA-IgA ≥1:10 was related to inferior OS with statistical significance (*p* = 0.002), and was related to poorer PFS with borderline significance (*p* = 0.082). Serum VCA-IgA ≥1:160 was significantly related to inferior OS and PFS (*p* < 0.0001 and *p* = 0.003, respectively) (Fig. 4).

**Fig. 5:**
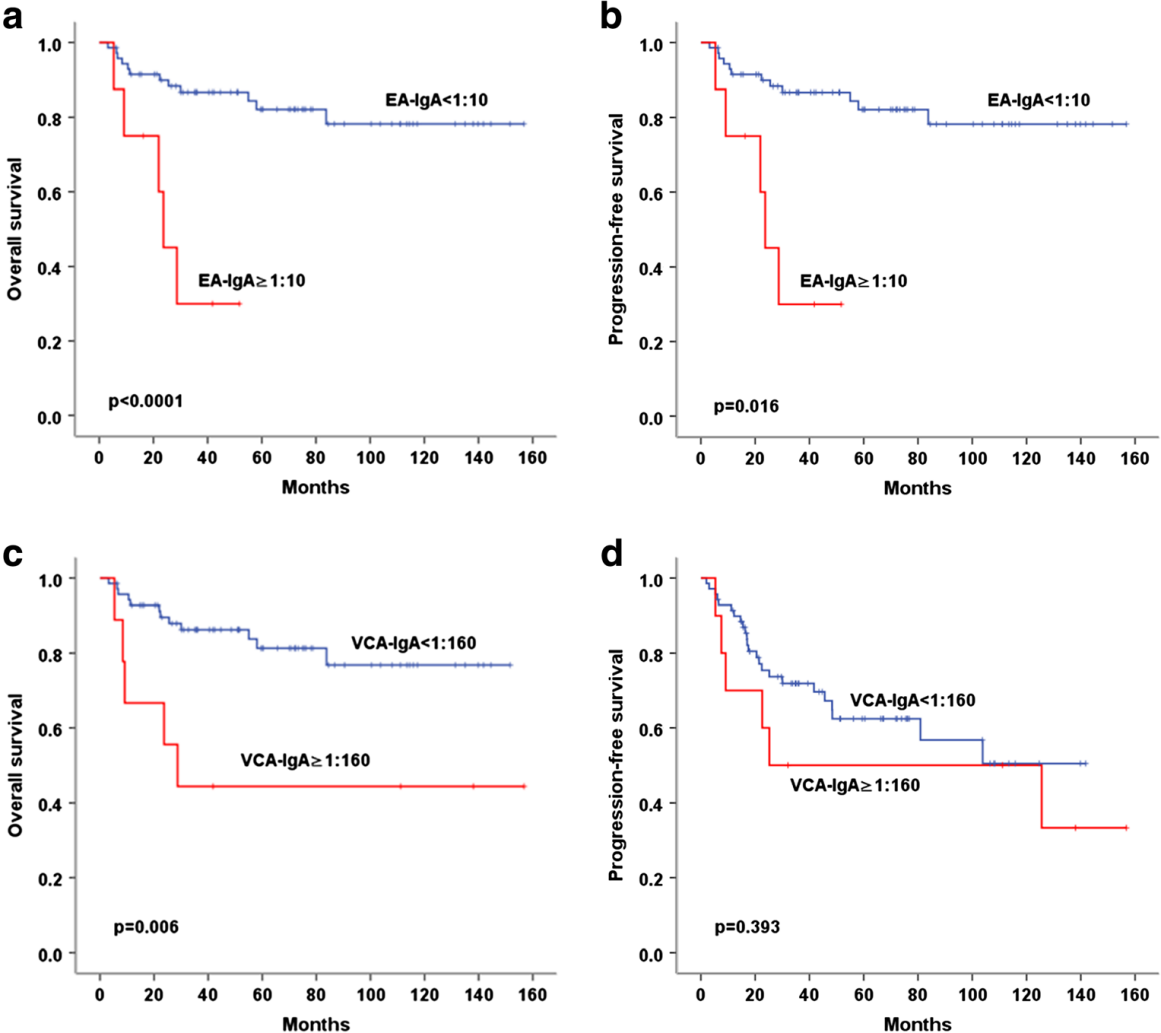
Comparison of OS and PFS in CR patients according to serum EA-IgA and VCA-IgA level. **a**, **b** EA-IgA ≥1:10 correlated with significantly inferior OS and PFS in patients who got CR after treatment. **c**, **d** Serum VCA-IgA ≥1:160 significantly correlated with inferior OS in CR patients, but not PFS

Of the 80 patients who got CR, these with serum EA-IgA ≥1:10 had significantly inferior PFS and OS than those with serum EA-IgA <1:10 (*p* < 0.0001 and *p* = 0.016, respectively). Patients with serum VCA-IgA ≥1:160 also had inferior OS than those with VCA-IgA <1:160 (*p* = 0.006), but no statistical significance was found for PFS (*p* = 0.393) (Fig. 5). On the contrary, both EA-IgA and VCA-IgA failed to predict the outcome of the patients who did not get CR.

To explore further prognostic impact of serum EA-IgA and VCA-IgA in patients with different treatment modalities, follow-up data were examined according to serum EA-IgA and VCA-IgA level in patients with different treatment modalities using Kaplan-Meier analysis and the log-rank test. In patients who had chemotherapy followed by radiotherapy, serum EA-IgA ≥1:10 was significantly related to inferior OS and PFS (*p* = 0.003 and *p* = 0.032, respectively) (Fig. S3); while serum VCA-IgA ≥1:160 was only related to poorer OS with borderline significance (*p* = 0.076). In patients who had chemotherapy alone, serum VCA-IgA ≥1:160 was significantly related to inferior OS (*p* = 0.042). Neither EA-IgA ≥1:10 nor VCA-IgA ≥1:160 were found related to OS or PFS in patient had chemotherapy alone.

### Combination of EBV serology and plasma EBV DNA to predict outcome in ENKTCL patients

**Fig. 6:**
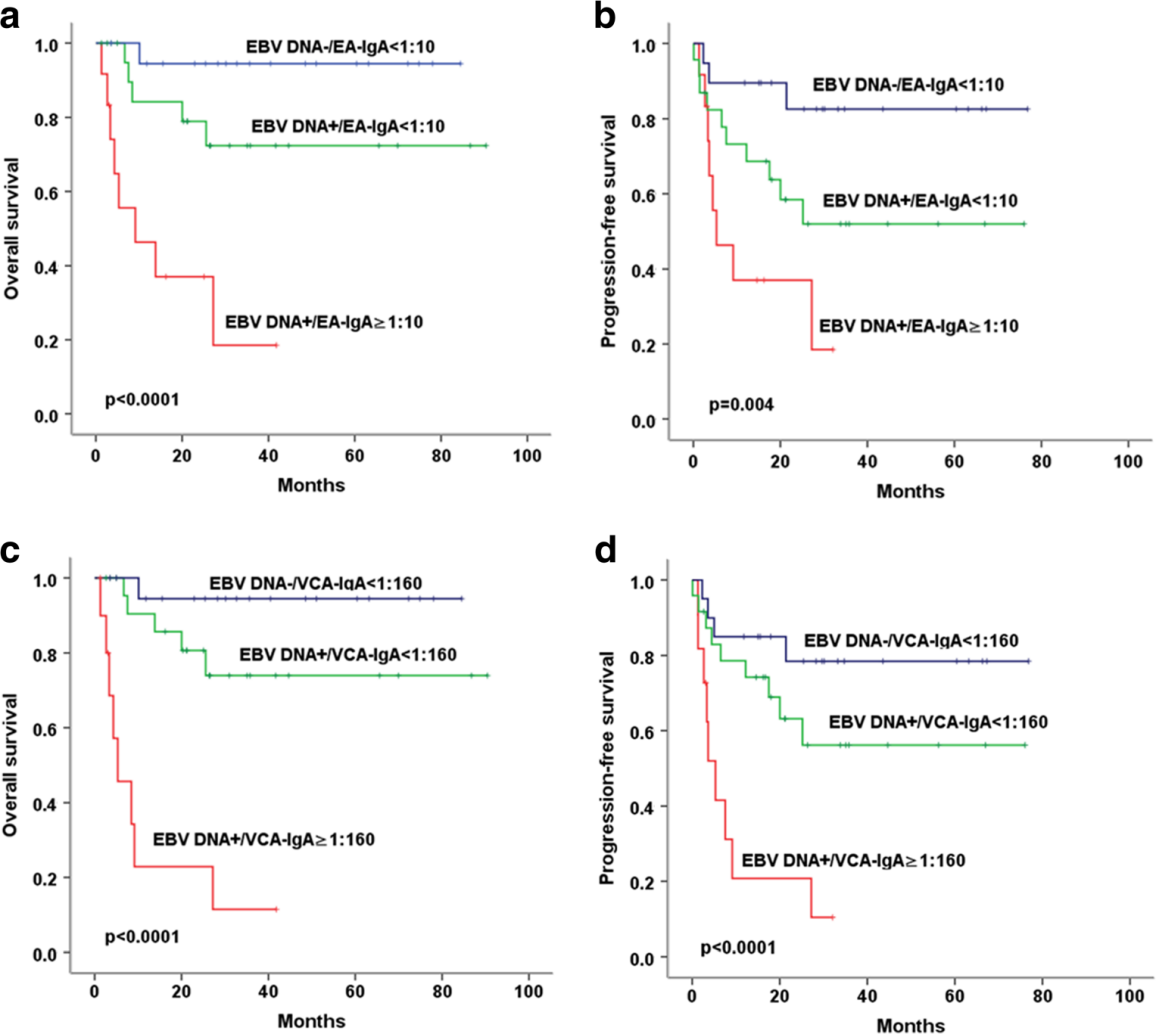
Comparison of OS and PFS according to plasma EBV DNA load and serum level of EA-IgA and VCA-IgA. **a**, **b** The periods of OS and PFS in patients with EA-IgA ≥1:10/EBV DNA+ were significantly shorter than that of patients with EA-IgA <1:10/EBV DNA+ and EA-IgA <1:10/EBV DNA−. **c**, **d** The periods of OS and PFS in patients with VCA-IgA ≥1:160/EBV DNA+ were significantly shorter than that of patients with VCA-IgA ≥1:160/EBV DNA+ and VCA-IgA <1:160/EBV DNA−

According to serum EA-IgA and plasma EBV DNA level, patients with ENKTCL were divided into the following three groups: EA-IgA ≥1:10/EBV DNA+, EA-IgA <1:10/EBV DNA+, and EA-IgA <1:10/EBV DNA−. Further analyses revealed that the periods of OS and PFS in patients with EA-IgA ≥1:10/EBV DNA+ were significantly shorter than that of patients with EA-IgA <1:10/EBV DNA+ and EA-IgA <1:10/EBV DNA− (*p* < 0.0001 and *p* = 0.003, respectively). Similarly, the periods of OS and PFS in patients with VCA-IgA ≥1:160/ EBV DNA+ were also significantly shorter than that of patients with VCA-IgA <1:160/EBV DNA+ and VCA-IgA <1:160/EBV DNA− (both *p* < 0.0001). Accordingly, patients with both EBV DNA positive and EA-IgA ≥1:10 or VCA-IgA ≥1:160 had the highest risk for ENKTCL progression and mortality (Fig. 6).

## Discussion

To our best of knowledge, this is the largest cohort study to explore the manifestation of serum EBV EA-IgA and VCA-IgA antibodies in ENKTCL. Of interesting, although EBV could be detected by EBER in situ hybridization in tumor cells for all the cases in our series, only 18.4 and 41.1% of ENKTCL patients showed positivity for serum EBV EA-IgA and VCA-IgA, respectively. Therefore, there is a lack of correlation between EBV serology and presence of EBV in the tumor cells of patients with ENKTCL, similar to earlier findings in Hodgkin lymphoma [29]. This suggests that serum EA-IgA and VCA-IgA cannot be used for assisting in diagnosis and for screening of ENKTCL. In addition, our data demonstrated a close relationship between high-level EBV serology and adverse clinical characteristics. Both serums EA-IgA ≥1:10 and VCA-IgA ≥1:160 were more frequently observed in ENKTCL patients with advanced stage disease, IPI score ≥2, regional lymph node involvement, and extranodal involvement sites >1. This observation reveals that high level of EA-IgA and VCA-IgA associated with the aggravation of ENKTCL. The biologic mechanism is unclear at present. One possibility is that high level of lytic antibodies indicates a lytic phase of EBV infection leading to the proliferation of EBV and the overexpression of EBV-associated oncoproteins. These oncoproteins may, thus, result in further transformation of the tumor cells into more aggressive clones. The tumor cells of ENKTCL contain EBV virus gene as well as a variety of EBV-specific antigens, such as EBV EA and VCA. Thus, serum EA-IgA and VCA-IgA antibodies could be detected. The other possible mechanism is that EBV latently infects human B cells in more than 90% of normal population. Aggravation of the disease active EBV that let the virus enter the replication phase, resulting lytic antibodies, such as EBV EA-IgA and VCA-IgA, could be detected. Serum EA-IgA and VCA-IgA level in patients with ENKTCL may indirectly reflect the host’s immune status or EBV physiological state in the body.

Prior to this study, the prognostic value of EBV serology in ENKTCL has not been reported. The present study firstly explored the value of serum EA-IgA and VCA-IgA in patients with ENKTCL, which showed a significant association with survival outcomes. Both EA-IgA ≥1:10 and VCA-IgA ≥1:160 were significantly related to inferior OS and PFS in patients with ENKTCL. Multivariate analysis also indicted that EA-IgA ≥1:10 was an independent prognostic factor for OS. Of note, VCA-IgA positivity (≥1:40) had no significant correlation with clinical features or prognosis, except for significant correlation with low-CR rate (Table S1). The current study also suggested that serum EA-IgA is superior to VCA-IgA for risk group stratification and prognostic prediction in ENKTCL. Although patients with stage I/II ENKTCL showed better prognosis than patients with stage III/IV, there is still a lack of prognostic markers resulting significant challenges in treatment selection for the very heterogeneous clinical behavior. Our results demonstrated that serum EA-IgA and VCA-IgA level at diagnosis could distinguish those with poor outcomes from those with favorable outcomes in patients with stage I/II and IPI score 0–1. EA-IgA ≥1:10 significantly associated with poor PFS and OS; while VCA-IgA ≥1:160 significantly correlated to poorer OS in patients with stage I/II. Both EA-IgA ≥1:10 and VCA-IgA ≥1:160 were found to significantly affect OS in patients with IPI score 0–1. These findings suggest that serums EA-IgA and VCA-IgA were good markers for risk group stratification in patients with early stage group and low-IPI score. A simple and regular way might be established to identify ENKTCL patients of different risks at diagnosis.

As plasma EBV DNA has been reported to be a valuable biomarker of predicting early relapse for patients with early stage ENKTCL in the era of asparaginase [25], the finding prompted us to investigate the relationship of serum EA-IgA and VCA-IgA level and treatment response in ENKTCL patients. The current study confirmed the negative association between serum EA-IgA and VCA-IgA level and treatment response. In our study, patients with serum EA-IgA ≥1:10 had a significantly lower CR rate; while patients with serum VCA-IgA ≥1:160 seemed to have a lower CR rate with borderline significance. In addition, pretreatment serum EA-IgA ≥1:10 showed significant impact on subsequent tumor relapse. In recent years, many cancer centers have adopted the combination of chemotherapy and radiotherapy (primary chemotherapy followed by radiotherapy or concurrent chemoradiotherapy) for localized disease to reduce the relapse rate [30, 31]. However, is seems that no good biomarkers are defined yet to predict the response rate of the treatment. Of interest, the current study revealed that EA-IgA ≥1:10 significantly correlated with inferior OS and PFS in patients who had chemotherapy followed by radiotherapy. These findings indicate that pretreatment serum EA-IgA level may be a good candidate for the prediction of response after chemotherapy followed by radiotherapy. If validated in future perspective clinical trials, patients with pretreatment serum EA-IgA ≥1:10 may benefit from treatment strategy adjustment.

EBV DNA has been reported as a prognostic factor for the relapse and survival of ENKTCL patients [20, 24, 25]. Our study demonstrated a close correlation in ENKTCL patients between serum EA-IgA and VCA-IgA level and clinical outcomes. These findings prompted us to determine the value of combination of serum EBV antibodies with plasma EBV-DNA in predicting the survival of patients with ENKTCL. Similar to earlier findings showing higher positivity rate of EBV DNA than serum VCA-IgA antibody in NPC patients [11, 12, 32], higher positivity rate was observed for EBV DNA (61.4%) than VCA-IgA (41.1%) and EA-IgA (18.4%) in ENKTCL patients. We compared the periods of OS and PFS among these 57 patients with EBV DNA data. Of interest, we found that patients with positivity of EBV DNA and EA-IgA ≥1:10 had the worst OS and PFS while patients with negativity of EBV DNA and EA-IgA <1:10 had the longest period of OS and PFS among these patients. Patients with positive EBV DNA alone showed intermediate period of OS and PFS. Similar results were also showed when combined EBV DNA and VCA-IgA ≥1:160. Apparently, the risk for the progression and mortality of ENKTCL may range from patients with positivity for EBV DNA and EA-IgA ≥1:10 or VCA-IgA ≥1:160 and positivity for EBV DNA alone to negativity for EBV DNA and EA-IgA <1:10 or VCA-IgA <1:160. Contents of EBV DNA, together with EA-IgA or VCA-IgA, can stratify the ENKTCL patients into three distinct risk groups and are valuable for prognosis. Hence, simultaneous tests of plasma EBV DNA and serum serology (EA-IgA and VCA-IgA) are valuable to identify the ENKTCL patients at high risk for the progression and mortality. Accordingly, we should closely follow up those patients with positivity for both measures to early detect and treat the recurrence of ENKTCL. Our findings may provide new references for clinical practice.

The current study had some limitations. Although a large cohort of ENKTCL patients was included, the numbers of cases in some subgroups were still small and some of the results may require verification using a larger cohort. Furthermore, due to its retrospective design and selection, information bias is possible. Also, all patients included were ethnically Chinese, which may limit the ability to extrapolate our findings to other populations. Further investigation is warranted to provide a better understanding of the mechanisms underlying the relation between EBV serology and ENKTCL.

In conclusion, high levels of pretreatment serum EBV EA-IgA and VCA-IgA antibodies were related to adverse ENKTCL profile, poor treatment response, early relapse, and poor outcome. In addition, these markers also could distinguish those with poor outcomes from those with favorable outcomes in patients with early stage, low-IPI score, positive pretreatment EBV DNA, and who had chemotherapy followed by radiotherapy. Furthermore, patients with both EBV DNA positive and high level of EA-IgA or VCA-IgA had a significantly higher risk for the progression and mortality. Simultaneous tests of both EBV serology and plasma EBV DNA may be valuable for the prognosis of ENKTCL in the clinic. These results indicated that high levels of serum EBV EA-IgA and VCA-IgA antibodies can be used for risk group stratification and prognostic prediction in ENKTCL.

## Electronic supplementary material

Table S1 (DOCX 26 kb)

Figure S1 (DOCX 2481 kb)

Figure S2 (DOCX 152 kb)

Figure S3 (DOCX 145 kb)
