# Supplementary material for: Serum EBV EA-IgA and VCA-IgA antibodies can be used for risk group stratification and prognostic prediction in extranodal NK/T cell lymphoma: 24-year experience at a single institution
Source: Ann Hematol. 2017 May 27;96(8):1331–42. doi: 10.1007/s00277-017-3013-y (PMC5486802; doi:10.1007/s00277-017-3013-y)
Supplement: Supplementary file 1 — (DOCX 26 kb) [file 277_2017_3013_MOESM1_ESM.docx]

**Table S1. Pretreatment serum EBV VCA-IgA and EA-IgA status of patient with NKTCL stratified by clinicopathological features**

|  |  | EA-IgA |  |  |  | VCA-IgA |  |
| --- | --- | --- | --- | --- | --- | --- | --- |
| **Characteristics** | <1:10  n (%) | ≥1:10  n (%) | p-value |  | <1:40  n (%) | ≥1:40  n (%) | p-value |
| Patients | 115（81.6） | 26（18.4） |  |  | 82(58.2) | 59(41.8) |  |
| **Age** |  |  |  |  |  |  |  |
| ≤60 | 98(83.1) | 20(16.9) | 0.301 |  | 71(60.2) | 47(39.8) | 0.272 |
| >60 | 17(73.9) | 6(26.1) |  |  | 11(47.8) | 12(52.2) |  |
| **Gender** |  |  |  |  |  |  |  |
| Male | 81(82.7) | 17(17.3) | 0.613 |  | 60(61.2) | 38(38.8) | 0.256 |
| Female | 34(79.1) | 9(20.9) |  |  | 22(51.2) | 21(48.8) |  |
| **Annabor Stage** |  |  |  |  |  |  |  |
| Ⅰ/Ⅱ | 107(84.9) | 19(15.1) | **0.008** |  | 76（60.3） | 50(39.7) | 0.132 |
| Ⅲ/Ⅳ | 8(53.3) | 7(46.7) |  |  | 6(40.0) | 9(60.0) |  |
| **B symptoms** |  |  |  |  |  |  |  |
| Absence | 61(87.1) | 9(12.9) | 0.075 |  | 45(64.3) | 25(35.7) | 0.148 |
| Presence | 52(75.4) | 17(24.6) |  |  | 36(52.2) | 33(47.8) |  |
| **LDH** |  |  |  |  |  |  |  |
| Normal | 87（83.7） | 17（16.3） | 0.190 |  | 61（58.7） | 43（41.3） | 0.776 |
| Elevated | 25（73.5） | 9（26.5） |  |  | 19（55.9） | 15（44.1） |  |
| **IPI score** |  |  |  |  |  |  |  |
| 0-1 | 80（86.0） | 13（14.0） | **0.036** |  | 57（61.3） | 36（38.7） | 0.256 |
| 2-5 | 32（71.1） | 13（28.9） |  |  | 23（51.1） | 22（48.9） |  |
| **ECOG PS score** |  |  |  |  |  |  |  |
| 0-1 | 103（82.4） | 22(17.6) | 0.393 |  | 73(58.4) | 52（41.6） | 0.605 |
| 2-5 | 7（70.0） | 3(30.0) |  |  | 5（50.0） | 5（50.0） |  |
| **Primary tumor site** |  |  |  |  |  |  |  |
| UAT | 109(81.3) | 25(18.7) | 1.000 |  | 77(57.5) | 57(42.5) | 0.465 |
| Non-UAT | 6(85.7) | 1(14.3) |  |  | 5(71.4) | 2(28.6) |  |
| **Regional LN involvement** |  |  |  |  |  |  |  |
| No | 71(92.2) | 6(7.8) | **0.000** |  | 48(62.3) | 29(37.7) | 0.270 |
| Yes | 44(68.8) | 20(31.3) |  |  | 34(53.1) | 30(46.9) |  |
| **Extranodal sites** |  |  |  |  |  |  |  |
| <2 | 105(84.0) | 20(16.0) | **0.035** |  | 75(60.0) | 50(40.0) | 0.322 |
| ≥2 | 9(60.0) | 6(40.0) |  |  | 7(46.7) | 8(53.3) |  |
| **Pretreatment EBV-DNA** |  |  |  |  |  |  |  |
| negative | 22(100.0) | 0(0.0) | **0.002** |  | 15(68.2) | 7(31.8) | 0.097 |
| positive | 23(65.7) | 12(34.3) |  |  | 16(45.7) | 19(54.3) |  |
| **Treatment response** |  |  |  |  |  |  |  |
| CR | 72(90.0) | 8(10.0) | **0.001** |  | 53(66.3) | 27(33.8) | **0.032** |
| Non-CR | 30(66.7) | 15(33.3) |  |  | 21(46.7) | 24(53.3) |  |

B symtoms include unexplained fever with temperature above 38℃,night sweating or weight loss more than 10% within 6 monthes; LDH, lactate dehydrogenase; IPI, International Prognostic Index; ECOG PS, Eastern Cooperative Oncology Group performance status; UAT, upper aerodigestive tract; CR, complete response.
