# Supplementary material for: Serum EBV EA-IgA and VCA-IgA antibodies can be used for risk group stratification and prognostic prediction in extranodal NK/T cell lymphoma: 24-year experience at a single institution
Source: Ann Hematol. 2017 May 27;96(8):1331–42. doi: 10.1007/s00277-017-3013-y (PMC5486802; doi:10.1007/s00277-017-3013-y)
Supplement: Supplementary file 2 — (DOCX 2481 kb) [file 277_2017_3013_MOESM2_ESM.docx]

**
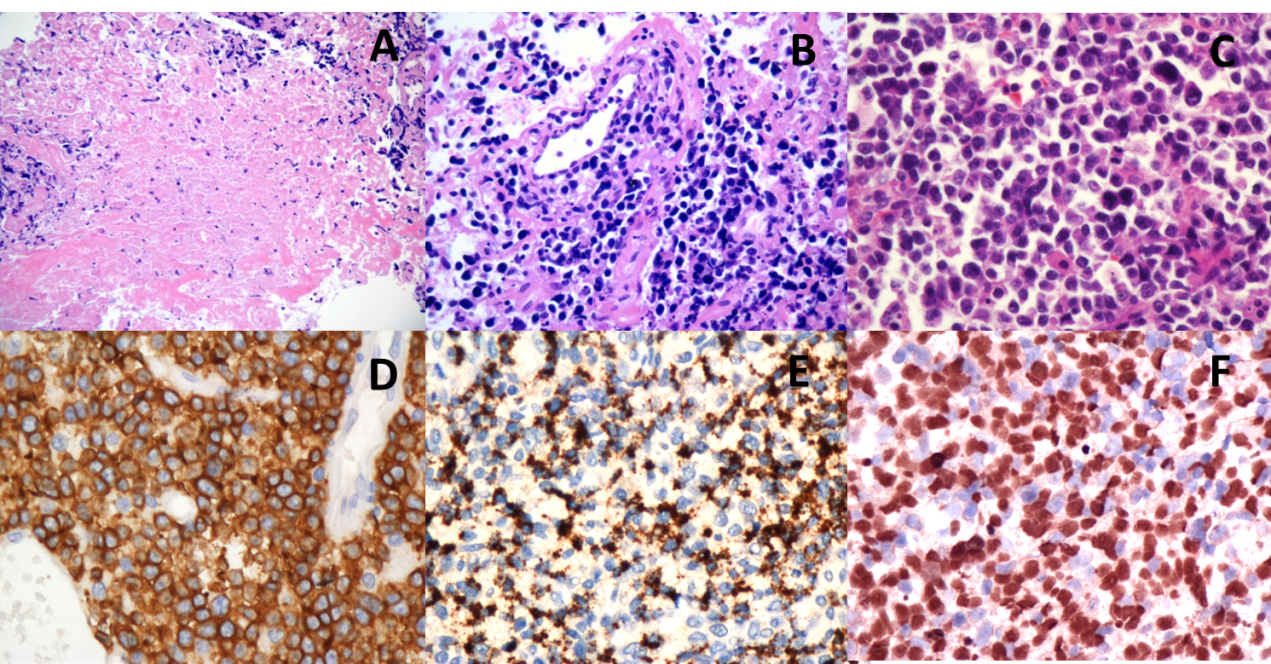
**

**Figure S1.** Morphology and immunophenotype of extranodal NK/T-cell lymphoma

1. Extensive coagulative necrosis was found within the tumor (H&E, ×100); B. An angiocentric and angiodestructive growth pattern was present (H&E, ×200); C. Tumor cells were of variable size, with irregularly folded nuclei (H&E, ×400); D. The lymphoma cells exhibited cytoplasmic CD3 (IHC×400); E. The tumor cells showed granular staining for TIA1 (IHC×400); F, EBV were detected in the nuclei in almost all the neoplastic cells by in situ hybridization for EBV-encoded RNA (EBER) (ISH×400).
