# Supplementary material for: Serum EBV EA-IgA and VCA-IgA antibodies can be used for risk group stratification and prognostic prediction in extranodal NK/T cell lymphoma: 24-year experience at a single institution
Source: Ann Hematol. 2017 May 27;96(8):1331–42. doi: 10.1007/s00277-017-3013-y (PMC5486802; doi:10.1007/s00277-017-3013-y)
Supplement: Supplementary file 3 — (DOCX 152 kb) [file 277_2017_3013_MOESM3_ESM.docx]

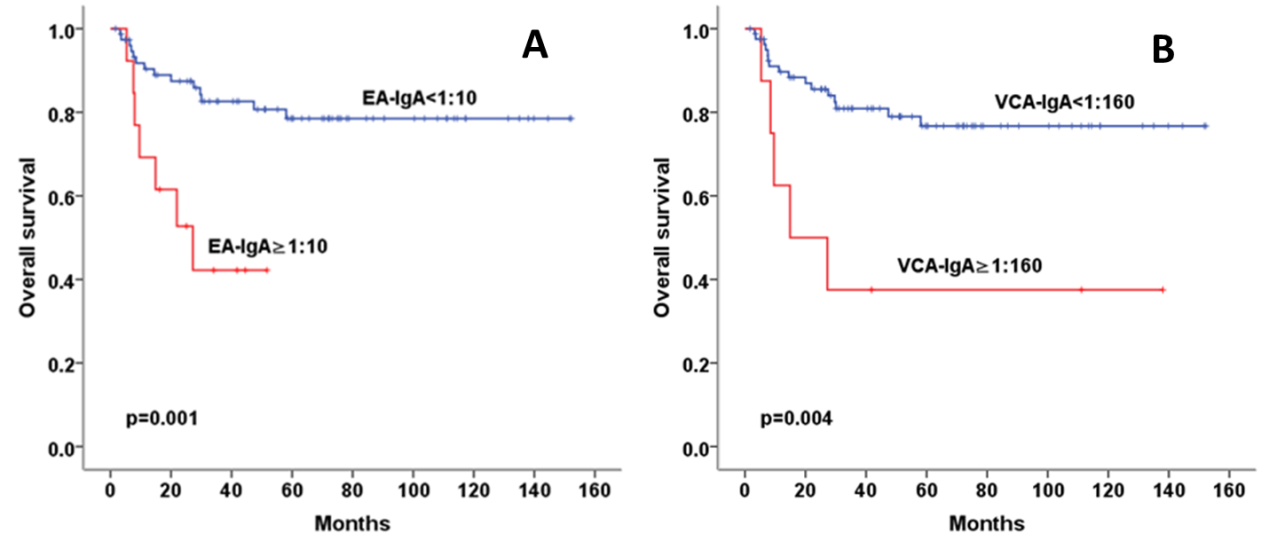


**Figure S2.** Comparison of OS and PFS in patients with IPI score 0-1 according to serum EA-IgA and VCA-IgA level.

1. EA-IgA ≥1: 10 was found to significantly affect OS in patients with IPI score 0-1; B. VCA-IgA ≥1: 160 significantly associated with inferior OS in patients with IPI score 0-1.
