# Supplementary material for: Serum EBV EA-IgA and VCA-IgA antibodies can be used for risk group stratification and prognostic prediction in extranodal NK/T cell lymphoma: 24-year experience at a single institution
Source: Ann Hematol. 2017 May 27;96(8):1331–42. doi: 10.1007/s00277-017-3013-y (PMC5486802; doi:10.1007/s00277-017-3013-y)
Supplement: Supplementary file 4 — (DOCX 145 kb) [file 277_2017_3013_MOESM4_ESM.docx]

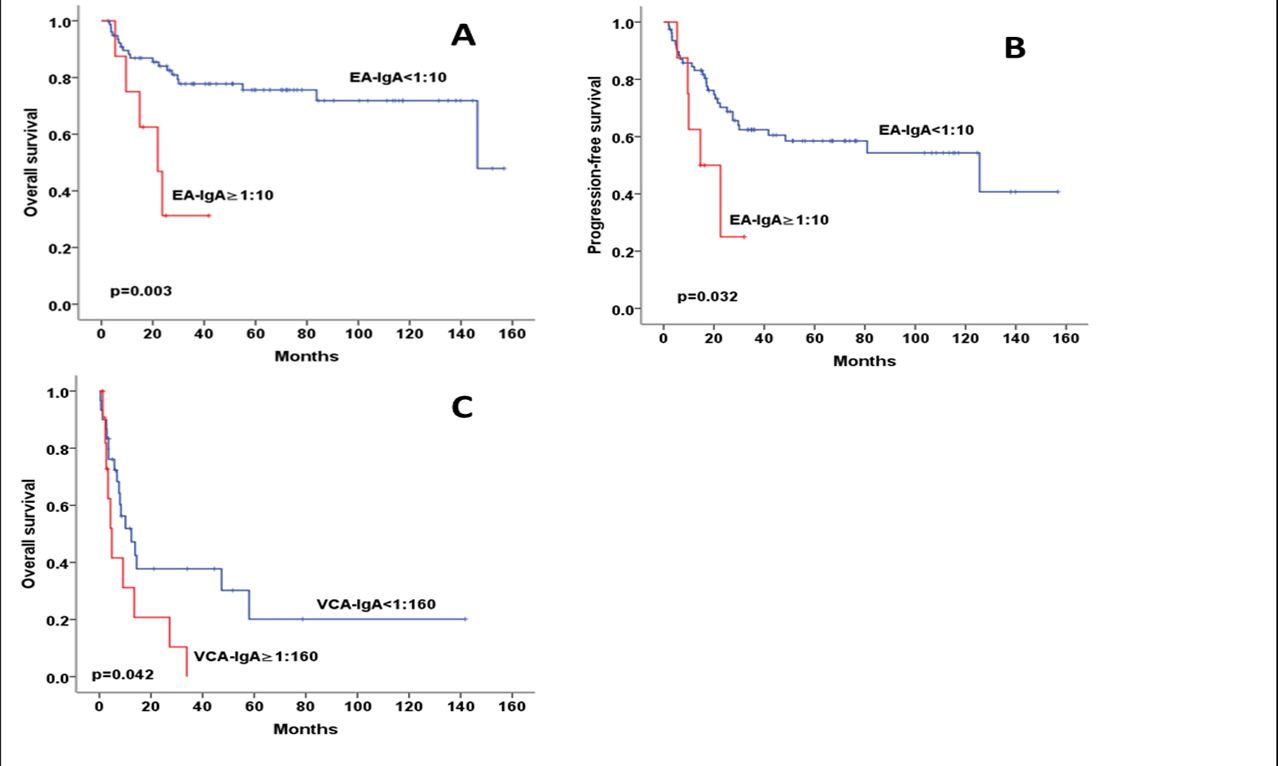


**Figure S3**. Subgroup survival analysis according to treatment modalities

A,B. In patients who had chemotherapy followed by radiotherapy, EA-IgA ≥1: 10 correlated with significantly inferior OS and PFS; C. In patients who had chemotherapy alone, VCA-IgA ≥1: 160 correlated with significantly inferior OS.
